# Supplementary material for: Facilitating factors and barriers in help-seeking behaviour in adolescents and young adults with depressive symptoms: A qualitative study
Source: PLoS One. 2021 Mar 8;16(3):e0247516. doi: 10.1371/journal.pone.0247516 (PMC7939362; doi:10.1371/journal.pone.0247516)
Supplement: Attachment — Submitted filename: Response to Reviewers.docx [file pone.0247516.s002.docx]

Prof. dr. T. van Amelsvoort
Academic Editor
PLOS ONE

February 5, 2021

Subject: Revision *“Facilitating factors and barriers in help-seeking behaviour in adolescents and young adults with depressive symptoms: a qualitative study”* (PONE-D-20-29462R1).

Dear prof. dr. van Amelsvoort,

Thank you for reviewing our manuscript and for the opportunity to submit a revision. We appreciate the feedback from Reviewer #2 and have taken it into consideration.

Although we intend to comply with the requests of Reviewer #1 and Reviewer #2, we feel that the remarks about the integration of the conceptual framework are difficult to address in such a way that does justice to the suggestions and concerns of both reviewers.

After deliberation in our research group, we have chosen to integrate the framework as described in the Response to reviewers below. Hopefully, you will appreciate our proposed revision.

We believe that the manuscript has improved by incorporating the reviewers’ suggestions and hope you will consider it for publication in PLOS ONE.

Please feel free to contact us in case further clarifications are needed.

On behalf of all co-authors,

Yours sincerely,

Eline Eigenhuis and Ruth C. Waumans

Amsterdam UMC, location VUmc, Department of Psychiatry

GGZ inGeest

Oldenaller 1, 1081 HJ Amsterdam, The Netherlands.

E-mail: [e.eigenhuis@ggzingeest.nl](mailto:e.eigenhuis@ggzingeest.nl)

Response to Reviewer #2:

Reviewer #2: I thank the authors for their thorough and helpful response letter. The discussion of additional research in the introduction is helpful, and the additions to the limitations and implications sections strengthen the paper. However, I have some concerns about the conceptual integrity and clarity of the paper as currently presented, and believe further clarifications are needed.

Reviewer #1 suggested that “The [five help-seeking] themes could be strengthened by using (and adding to) a conceptual help-seeking framework (eg, Rickwood et al 2012) so that the terminology and findings can be compared across studies.”

In response to this suggestion, the authors now state that the study was informed by the help-seeking framework by Rickwood and Thomas (2012):

“In terms of the conceptual measurement framework from Rickwood and Thomas (22), this study focused on all three Process aspects (Orientation, Intention and Behaviour), in a broad time frame (Ever), from Formal help sources (Source), in participants with a specific syndrome type, i.e. depressive symptoms (Concern). Information on all types of help (Type) was gathered, with a specific focus on information and treatment.”

However, it remains unclear how exactly the framework did inform the paper. The authors state that the study focused “on all three Process aspects of help-seeking behaviour (Orientation, Intention and Behaviour)” (p. 22, line 442), but there is no explicit discussion of these three process aspects either in the findings or discussion section, and they also do not appear in the coding frame (Table 2). More generally, it does not seem that the authors used the framework to structure the discussion of findings, or their contextualisation within existing literature, as suggested by reviewer #1. As such, the Rickwood framework appears to be an afterthought, rather than a conceptual backbone. I might suggest that the authors engage with the framework more thoroughly and meaningfully, in line with the suggestions made by Reviewer #1. Alternatively, the authors could consider removing reference to the framework altogether. I might avoid a middle ground that may appear tokenistic and could undermine the paper’s credibility. If the authors decide to maintain reference to the conceptual framework, it would be helpful to (a) introduce its key constructs and dimensions in the introduction, (b) clarify that it did not inform the study design (e.g., design of the topic guide; design of the coding frame), (c) state more explicitly how it informed the paper.

Response:	We thank the reviewer for this important feedback. We endorse the remark from the reviewer that the framework has not been used to structure our findings or to form the coding tree, and as such was not part of the design of our study. We do however value the suggestion of Reviewer #1 to use a conceptual framework to structure findings in studies on help-seeking. After thorough discussion within our research group on how to follow up on the suggestions of both reviewers, we came to the conclusion that integrating the framework in this phase of the study does not match with the inductive design of our study. Therefore, we decided to remove the text about the framework from the Methods section.

However, we acknowledge the importance of conceptual consistency in order to enable comparison of findings across studies using a conceptual framework such as proposed by Rickwood and Thomas. The following text has therefore been added to the Discussion section (page 26):

*“Previous research has shown that the literature on help-seeking behaviour comprises a variety of studies with different methodology, focus and outcomes, hampering direct comparison (22). In order to improve conceptual consistency, Rickwood and Thomas (22) presented a framework consisting of five aspects of help-seeking behaviour: Process (comprising the aspects of Orientation, Intention, Behaviour), Timeframe (i.e. past/next 4 weeks, past/next 12 months, Ever), Source (Formal help, Semi-formal help, Informal help, Self-Help), Type of help (Instrumental, Information, Affiliative, Emotional, Treatment) and Concern (General distress, Specific symptom types). Although we did not include this framework in the design of our study, our study can be categorised as focusing on all three Process aspects (Orientation, Intention and Behaviour) of help-seeking, in a broad time frame (Ever), from Formal help sources (Source), in participants with a specific syndrome type, i.e. depressive symptoms (Concern). Information on all types of help (Type) was gathered, with a specific focus on information and treatment. Future studies on help-seeking may use a conceptual framework such as described by Rickwood and Thomas (22) to increase homogeneity in study designs and comparability of the results.”*

In the Methods, the authors have helpfully expanded on the coding process. In addition, it would be helpful to state explicitly whether the coding process and creation of the thematic map was inductive, or informed by an existing coding frame (such as the framework by Rickwood and Thomas for example).

Response:	In order to clarify the process of data analysis, the text in the Methods section has been altered (page 7):

*“Data analysis was conducted in an inductive manner using thematic analysis (41), focusing on participants’ perceived barriers and facilitators in treatment-seeking.”*

At the start of the findings section, it might be helpful to introduce the five key help-seeking themes identified by the authors, rather than focusing just on the distinction between processes centred within the individual and externally focused processes. It is not entirely clear what value this additional taxonomic layer adds, in addition to the five help-seeking themes, and how this layer relates to the process aspects described by the Rickwood and Thomas framework (i.e., Orientation, Intention and Behaviour). Clarification would be helpful.

Response:	We agree with the reviewer that the focus of the start of the results section should be on the five main themes and their introduction. The distinction between internal and external processes was therefore removed from our text and from Table 2. The text now reads (page 10):

*“Analysis of the interviews generated five main themes with different barriers and facilitators for help-seeking. These main themes were (I) Individual functioning and well-being, (II) Health literacy, (III) Attitudinal aspects, (IV) Surroundings, and (V) Accessibility. A complete overview of the findings can be found in Table 2. The most important themes are explained below.”*

Table 3 contains reference to Moroccan parents. The authors may want to adjust to “North Africa” for consistency with the remainder of the paper.

Response:	We thank the reviewer for noticing this inconsistency. We changed the text in Table 3 to ‘parents originating from Northern Africa’.

In several parts of the discussion (twice on page 23), the authors now state that “individual functioning and wellbeing – such as poor academic performance, physical symptoms, and mental distress – was often a prompt for help-seeking". The authors may want to clarify that it was impairment or a deterioration in functioning and wellbeing that prompted help-seeking.

Response:	We agree that the text could be clarified at this point. We added ‘impairment or a deterioration’ in two sentences of the discussion. The text now reads as follows (pages 22 & 23):

*“Impairment or deterioration in individual functioning and well-being– such as poor academic performance, physical symptoms, and mental distress – was often a prompt for help-seeking, whereas good academic performance despite depressive symptoms could be a hindrance.”*

And:

*“Our finding that impairment or deterioration in individual functioning and well-being prompt treatment-seeking is in accordance with previous research in adolescents (24,32,44)”*
